# Splenorenal bypass in suprarenal aneurysm with solitary left kidney: a case report

**DOI:** 10.1590/1677-5449.202500842

**Published:** 2025-11-28

**Authors:** Utkarsh Anand, Gokulakrishnan Hari, Vishesh Gupta, Varsha Khandelwal, Tanuj Singla, Ujjwal Gorsi, Ajay Savlania

**Affiliations:** 1 Postgraduate Institute of Medical Education and Research – PGIMER, Chandigarh, India.

**Keywords:** abdominal aortic aneurysm, solitary kidney, splenorenal, renal artery, aneurismas da aorta abdominal, rim único, esplenorrenal, artéria renal

## Abstract

Renal revascularization in patients with a solitary functioning kidney and a suprarenal abdominal aortic aneurysm (AAA) is a surgical challenge due to the risk of renal ischemia and postoperative renal dysfunction. We present a unique case in which a 69-year-old male with a solitary left kidney and suprarenal AAA was managed successfully with a preemptive splenic artery-to-left renal artery transposition (splenorenal bypass) followed by aneurysmorrhaphy. This approach preserved renal function and avoided ischemic insult during aortic reconstruction. At 2 years of follow-up, the patient maintains stable renal parameters and the reconstruction is patent, underscoring the utility of splenorenal bypass as a viable alternative in anatomically constrained and high-risk scenarios.

## Resumo

A revascularização renal em pacientes com rim solitário funcional e aneurisma de aorta abdominal (AAA) suprarrenal representa um desafio cirúrgico devido ao risco de isquemia e disfunção renal pós-operatória. Apresentamos o caso singular de um homem de 69 anos com rim esquerdo único e AAA suprarrenal, tratado com sucesso com anastomose preemptiva entre a artéria renal esquerda e a artéria esplênica (derivação esplenorrenal), seguida de aneurismorrafia. Essa abordagem preservou a função renal e evitou insulto isquêmico durante a reconstrução aórtica. Após 2 anos de acompanhamento, o paciente mantém parâmetros renais estáveis ​​e reconstrução pérvia, reforçando a utilidade da derivação esplenorrenal como alternativa viável em cenários anatomicamente restritos e de alto risco.

## INTRODUCTION

The global incidence of abdominal aortic aneurysm (AAA) is 0.92%.^1^ AAAs are classified based on their relation to the renal arteries, with infrarenal being the most common (80%), followed by juxtarenal (18%), while suprarenal AAAs are the least frequent, accounting for only 2% of cases.^2^ Patients with suprarenal AAAs have a higher incidence of renal artery stenosis (RAS), reported at 20%, compared to 5% in infrarenal cases.^3^ When unilateral RAS leads to renal atrophy, it may result in a solitary functioning kidney (SFK), which undergoes adaptive hyperfiltration to maintain overall renal function, a response that becomes maladaptive over time.^4^ This makes the SFK particularly vulnerable to renal deterioration during abdominal aortic aneurysm repair, as even short durations of aortic clamping in open surgery or contrast exposure and suprarenal fixation in endovascular repair can significantly increase the risk of acute kidney injury and progression to chronic kidney disease.^5,6^ We report a case of suprarenal AAA with an SFK, managed by preemptive splenic artery-to-left renal artery transposition prior to aortic clamping, with a two-year follow-up.

### Ethics statement/ethical approval

Ethics approval was not required for this case report, as per the policy of the Institutional Ethics Committee. The study complies with the ethical standards laid down in the 1964 Declaration of Helsinki and its subsequent amendments. Written informed consent was obtained from the patient for publication of the case details and accompanying images. A copy of the written consent is available from the corresponding author.

### Reporting guidelines

This case report was prepared in accordance with the CARE (CAse REport) guidelines developed by the CARE Group.

## CASE REPORT

**Figure 1:**
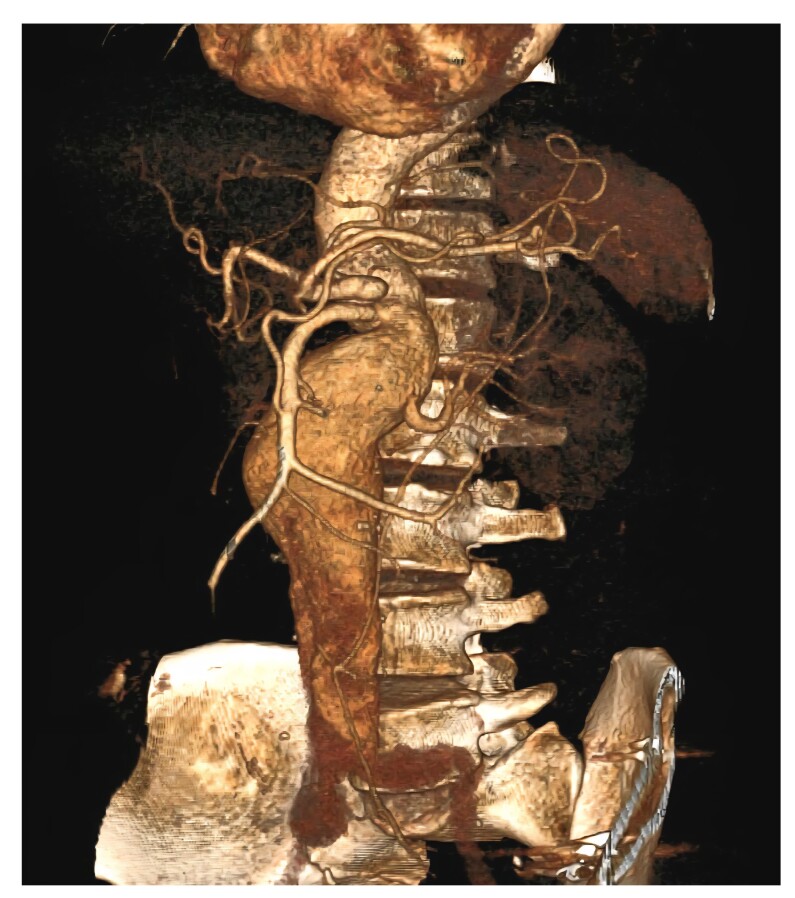
Preoperative 3D reconstructed CT angiography showing a fusiform dilatation of the abdominal aorta from L2 to L5 vertebral level with the left renal artery arising from the aneurysmal segment (suprarenal abdominal aortic aneurysm).

A 69-year-old man, with known comorbidities of hypertension and coronary artery disease, post coronary artery bypass grafting (CABG) in 2017, presented with abdominal pain of six months’ duration. The patient had a 42 pack-year smoking history and had stopped smoking five years previously. On examination, a pulsatile mass of size 10 × 8 cm was felt in the abdomen. All peripheral pulses were palpable. His blood investigations were within normal range, except for a reduced creatinine clearance of 42 mL/min, indicating moderate renal impairment. Computed tomography angiography revealed a suprarenal abdominal aortic aneurysm (AAA) with a maximum diameter of 56 mm and eccentric partial thrombosis (Figure 1). A solitary functional left kidney was present, with an atrophic right kidney due to atherosclerotic occlusion of the right renal artery. Renal scintigraphy demonstrated <10% function in the right kidney and >90% in the left. The patient’s estimated glomerular filtration rate (eGFR) was 54 mL/min/1.73 m^2^ preoperatively.

Based on these findings, the patient was planned to undergo open aneurysmorrhaphy combined with a splenic artery-to-left renal artery transposition. While endovascular aneurysm repair (EVAR) is the preferred approach in most patients with AAAs due to its minimally invasive nature and reduced perioperative morbidity, an open surgical approach was chosen in this case. The patient had good performance status and preserved left ventricular ejection fraction, making him a suitable candidate for open repair. At our institution, we follow an open-first strategy in anatomically favorable patients, particularly when socioeconomic factors limit access to advanced endovascular options. The patient was enrolled under the Government Health Scheme (Ayushman Bharat Scheme), which does not cover fenestrated or branched stent-grafts, restricting the feasibility of EVAR in this setting. Also, open repair offers superior long-term durability and avoids the use of nephrotoxic contrast agents, a critical consideration in patients with a solitary functioning kidney at increased risk for acute kidney injury.

A midline laparotomy with transperitoneal access revealed a fusiform aneurysm extending from the suprarenal aorta to the aortic bifurcation, measuring 10 × 8 cm and 15 cm in length. The right kidney was atrophic, while the left kidney appeared normal, with good pulsations in the left renal artery. A meticulous dissection of the left renal artery, splenic artery of sufficient length, proximal neck of the abdominal aortic aneurysm, and bilateral common iliac arteries was performed. Care was taken to preserve the short gastric vessels to maintain splenic viability. Prior to clamping of the splenic artery and the left renal artery, unfractionated heparin was administered at a dose of 1 mg/kg.

**Figure 2:**
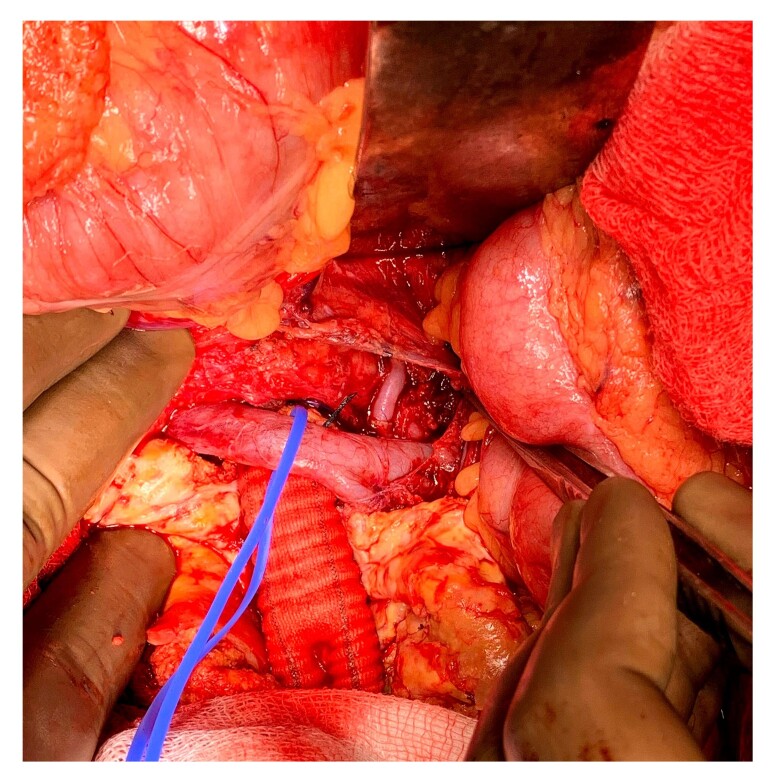
Intraoperative photograph demonstrating the splenorenal bypass (splenic artery to left renal artery). The blue vascular loop encircles the left renal vein, and the polyester graft is seen in the repaired aorta beneath.

**Figure 3:**
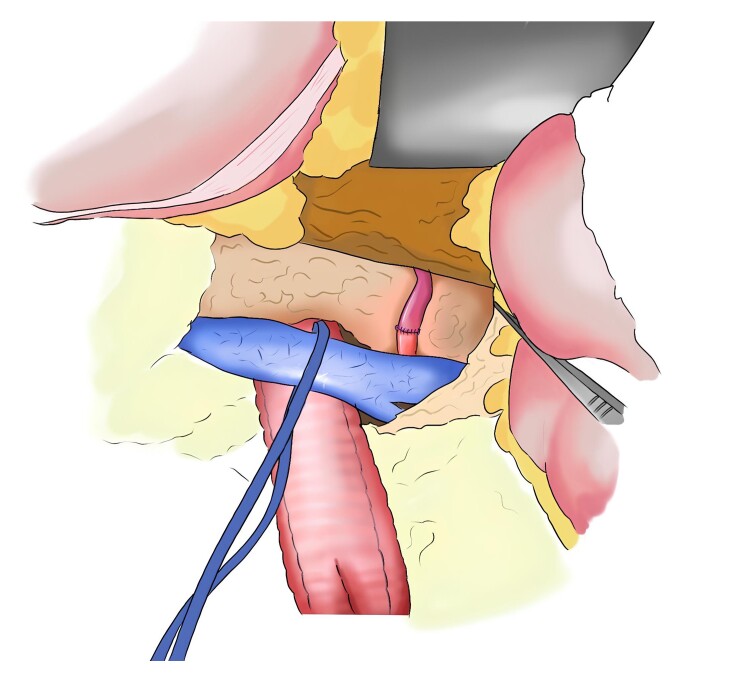
Schematic illustration showing the operative anatomy of a splenorenal bypass.

The procedure began with the splenorenal anastomosis, and definitive aortic reconstruction was planned for a later stage of the operation. The splenic artery was divided near the distal pancreas and routed in a tension-free manner retro-pancreatically to the left renal artery. The spleen was preserved, and a healthy, purple-colored spleen confirmed good vascularity. The left renal artery was divided and an end-to-end splenic artery-to-left renal artery anastomosis was performed using 6-0 polypropylene sutures with an anchoring technique (Figures 2 and 3). The total renal artery clamping time was less than 10 minutes, and good pulsatile flow in the renal artery was confirmed post-anastomosis.

Aortic reconstruction started with distal control at the iliac bifurcation and proximal control below the superior mesenteric artery (suprarenal control). The aneurysmal sac was evacuated, and a bifurcated polyester graft (16 × 8 mm) was used for reconstruction. End-to-end anastomosis was performed with 3-0 polypropylene sutures for inflow and 4-0 polypropylene sutures for outflow at the bilateral common iliac arteries. The aneurysm sac was closed over the graft using continuous 2-0 polydioxanone (PDS) sutures, followed by reperitonealization, and the rest of the abdomen was closed in the standard fashion.

**Figure 4:**
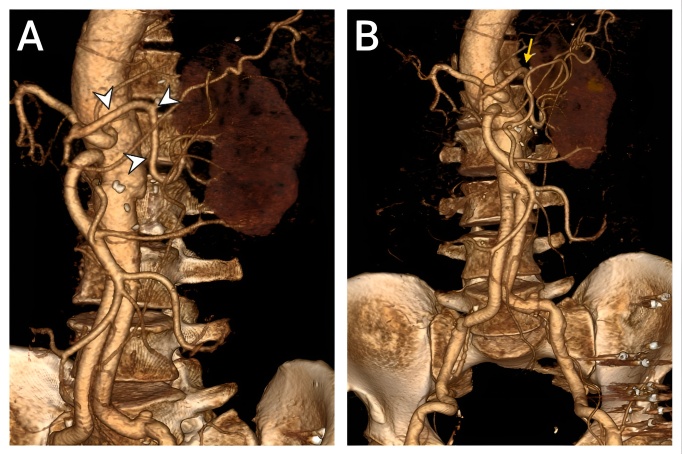
**A)** Postoperative 3D CT reconstruction in the left anterior oblique view demonstrates a patent splenorenal bypass (arrowheads). **B)** Anterior view shows the bypass (arrow) along with the reconstructed abdominal aorta.

Postoperatively, good flow in the left renal artery was confirmed with doppler. The patient was discharged on the 10th postoperative day with an eGFR of 38 mL/min/1.73 m^2^ and a creatinine clearance of 30 mL/min. At two-year follow-up, the patient remained well with a creatinine clearance of 46 mL/min. Follow-up CT angiography demonstrated an intact repair with a patent splenic artery-to-renal artery transposition (Figure 4).

## DISCUSSION

The first splenorenal anastomosis was done in 1952 by Thompson and Smithwick to treat hypertension due to left renal artery stenosis. The procedure failed, and the patient subsequently underwent a left nephrectomy 17 days later, following which his blood pressure normalized.^7^ Wylie, at the University of California, San Francisco, was among the first to advocate arterial autografts for renal revascularization.^8^ This work was later expanded by Stoney et al., who demonstrated the long-term efficacy and durability of iliac arterial autografts in a series of 94 patients undergoing renal artery reconstruction.^9^ These works established arterial autografts as a reliable and durable conduit. However, the use of renal revascularization procedures declined after the 1980s, primarily due to advances in antihypertensive pharmacotherapy, the widespread adoption of noninvasive imaging techniques, and growing evidence questioning the long-term benefits of revascularization for renovascular hypertension. Large trials like ASTRAL and CORAL further reinforced this trend by showing no significant advantage of stenting over optimal medical treatment.^10,11^

The current landscape of AAAs involving renal arteries revolves around EVARs with special adjuncts for renal arteries and open surgical repairs (OSR). Fenestrated EVAR (FEVAR) employs stent grafts with fenestrations that allow deployment of bridging stents for renal arteries. Chimney EVAR (Ch-EVAR) with endo-anchors has also been used to allow a parallel graft alongside the main aortic stent for renal arteries. If proximal approaches are not feasible, a retrograde approach can be used with the periscope technique that extends the renal artery stents downwards. Even though endovascular options have been shown to be safer than OSR in the perioperative period in multiple studies, they may not be suitable in patients with challenging anatomy.^12^

OSR for renal revascularization includes either a renal artery bypass using polyester or PTFE grafts, often pre-sewn to the aortic prosthesis to minimize ischemia time, or a renal artery reimplantation to the aorta. Both methods have been shown to have comparable long-term renal artery patency rates, with the choice between the two procedures dependent on the physician’s preference and local anatomy.^13^

A renal artery bypass to the splenic artery (splenorenal anastomosis) is an uncommon but valuable alternative in selected OSR cases where conventional techniques are not feasible.

Although the suprarenal aorta can serve as a proximal site for renal artery bypass, it carries drawbacks such as the need for aortic clamping, increased risk of ischemic injury to the kidneys, and technically demanding dissection.^14^ In contrast, the splenic artery provides a more accessible and less invasive inflow source, especially for the left renal artery. Splenorenal bypass has been successfully utilized as a rapid and effective salvage technique, particularly in scenarios such as bilateral renal artery occlusion during EVAR, where timely revascularization is critical to prevent permanent renal damage.^14^ Its utility is supported by long-term outcomes, with Khauli et al. reporting a 93% five-year patency rate in 69 patients who underwent splenorenal bypass for renal artery stenosis.^15^

An SFK compensates initially via glomerular hyperfiltration, but sustained increase in intraglomerular pressure leads to glomerular injury and nephron loss over time.^4^ Coexisting RAS adds chronic hypoperfusion, further accelerating damage. During suprarenal AAA repair, cross-clamping induces acute ischemia, creating a “triple-hit” of hyperfiltration, hypoperfusion, and ischemia. This combination significantly increases the risk of acute kidney injury and permanent renal loss unless protective strategies like renal revascularization are employed. Preoperative evaluation of the renal artery anatomy, renal perfusion and functional assessment, renal optimization, and multidisciplinary planning, along with good intraoperative surgical techniques, minimal renal artery clamping times, use of renoplegics, continuous urine output monitoring, and regular postoperative renal function monitoring are essential for managing such high-stakes situations.

## CONCLUSION

Splenorenal bypass remains a valuable open surgical option, particularly in patients with a solitary functioning left kidney and suprarenal aneurysm, in whom preemptive bypass may reduce renal failure-related morbidity and mortality. It is suitable in selected cases where endovascular or standard open techniques are not feasible, requiring careful assessment of anatomy, vascular dynamics, and overall patient status.

## Data Availability

All data generated or analyzed are included in this article and/or in the supplemental material.
